# Integrated intracellular metabolic profiling and pathway analysis approaches reveal complex metabolic regulation by *Clostridium acetobutylicum*

**DOI:** 10.1186/s12934-016-0436-4

**Published:** 2016-02-15

**Authors:** Huanhuan Liu, Di Huang, Jianping Wen

**Affiliations:** Key Laboratory of System Bioengineering (Tianjin University), Ministry of Education, Tianjin, 300072 People’s Republic of China; SynBio Research Platform, Collaborative Innovation Center of Chemical Science and Engineering (Tianjin), School of Chemical Engineering and Technology, Tianjin University, Tianjin, 300072 People’s Republic of China; TEDA Institute of Biological Sciences and Biotechnology, Nankai University, TEDA, Tianjin, 300457 People’s Republic of China; Key Laboratory of Molecular Microbiology and Technology, Ministry of Education, Tianjin, 300071 People’s Republic of China; Tianjin Key Laboratory of Microbial Functional Genomics, Tianjin, 300457 People’s Republic of China

**Keywords:** Butanol, Metabolic profiling analysis, Pathway analysis, *Clostridium acetobutylicum*, GC-MS, Metabolomics

## Abstract

**Background:**

*Clostridium acetobutylicum* is one of the most important butanol producing strains. However, environmental stress in the fermentation process usually leads to a lower yield, seriously hampering its industrialization. In order to systematically investigate the key intracellular metabolites that influence the strain growth and butanol production, and find out the critical regulation nodes, an integrated analysis approach has been carried out in this study.

**Results:**

Based on the gas chromatography-mass spectrometry technology, the partial least square discriminant analysis and the pathway analysis, 40 metabolic pathways linked with 43 key metabolic nodes were identified. In-depth analysis showed that lots of amino acids metabolism promoted cell growth but exerted slight influence on butanol production, while sugar metabolism was favorable for cell growth but unfavorable for butanol synthesis. Besides, both lysine and succinic acid metabolism generated a complex effect on the whole metabolic network. Dicarboxylate metabolism exerted an indispensable role on cell growth and butanol production. Subsequently, rational feeding strategies were proposed to verify these conclusions and facilitate the butanol biosynthesis. Feeding amino acids, especially glycine and serine, could obviously improve cell growth while yeast extract, citric acid and ethylene glycol could significantly enhance both growth and butanol production.

**Conclusions:**

The feeding experiment confirmed that metabolic profiling combined with pathway analysis provided an accurate, reasonable and practical approach to explore the cellular metabolic activity and supplied a basis for improving butanol production. These strategies can also be extended for the production of other important bio-chemical compounds.

**Electronic supplementary material:**

The online version of this article (doi:10.1186/s12934-016-0436-4) contains supplementary material, which is available to authorized users.

## Background

Bio-butanol, the next generation of liquid biofuels after bio-ethanol, has gained much interest due to many distinguished advantages, such as better blending ratio with gasoline, lower vapor pressure and corrosivity, higher energy density and less fuel consumption per unit [1, 2]. More importantly, as an ideal supplement or a sustainable replacement of gasoline, butanol can directly fuel the existing engines without any modification [1]. At present the production of butanol mainly depends on chemical method by propylene oxo synthesis in the industry [3]. However, serious environmental pollution, high oil prices and the exhaustion of oil resources force us to explore the sustainable clean alternative process for butanol production [4]. Hence, bio-butanol based on the classical acetone-butanol-ethanol (ABE) fermentation by microorganisms such as *Clostridium acetobutylicum*, *Clostridium beijerinckii* and similar strains, has been brought to light again [5].

However, in the last decade the butanol production has stayed at the level of approximately 10–20 g per liter during a batch ABE fermentation owing to some disadvantages by *C. acetobutylicum*. As a species of strictly anaerobic bacteria, *Clostridia* catabolizes a variety of sugars for cellular events, accompanying with the production of toxic metabolites, such as acetic acid, butyric acid, ethanol, butanol and acetone, which seriously inhibit cell growth and butanol production due to the acidified intracellular environment and the insufficient ATP production capacity [6], and even lead to the “acid crash” [7]. Moreover, the formation of endogenous spore can result in the termination of butanol secretion with a lower yield [8]. Butanol production, on the other hand, is limited by the defect of redox system, and the cell is highly sensitive to the redox status of intracellular and extracellular conditions [9–11]. Thus, it is critical to maintain high activity of the strain and improve the fermentation performance for butanol production.

Although the genomic annotation has implied the physiological conditions [12, 13] and several transcriptomics analysis have revealed the gene expression profiles [14–16], yet a lot of significant metabolic mechanisms remain obscure and a full understanding of the mechanism at systematic level has become increasingly important. In this study, we focused on the intracellular metabolic profiling and the pathway analysis of *C. acetobutylicum* to explore the effect of key metabolites on the strain growth and butanol synthesis systematically. Here, metabolomics was employed as an important technology for the quantitative analysis of critical metabolites and key reaction nodes by integrating the metabolic profiling and the computational tools including the partial least square (PLS) analysis and pathway analysis. The complex relationship between intercellular metabolites and fermentation characterization has been deciphered for the first time, thereby providing the key information for guiding the rational feeding strategy to efficiently improve microbial cell product yield.

## Results

### Fermentation profiles for cell growth and butanol production

**Fig. 1:**
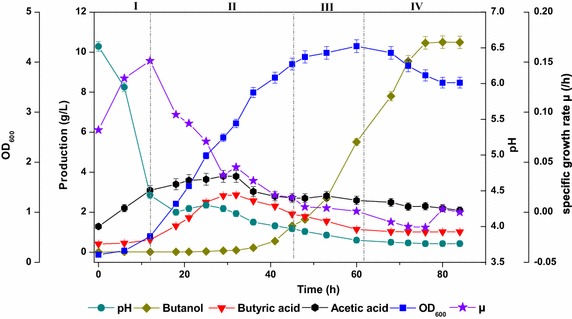
Fermentation profiles for *C. acetobutylicum* ATCC 824. The whole process could be divided into four phases based on cell growth, i.e., lag phase (0–12 h), log phase (12–45 h), stationary phase (45–63 h) and decline phase (63–84 h)

As shown in Fig. 1, the typical acidogensis period stretched from the lag to the mid-log phase (0–32 h), and the solventogensis spanned from the mid-log phase to stationary, and to the decline phase (32–76 h). In the lag phase, pH quickly decreased from 6.8 to 4.3 due to the formation of acetic acid accompanied by ATP synthesis through substrate level phosphorylation. In the log phase, pH firstly rose slightly to 4.4, and then gradually dropped down until the end of fermentation. From 12 to 32 h, the net secretion rate of acetic acid slowed down, while the butyric acid concentration increased. A small amount of butanol could be detected (~0.1 g/L) when the accumulation of butyric acid and acetic acid reached to the peak at 32 h. In addition, it’s worth noting that the specific cell growth rate began to flatten out when the production of butanol started. During the stationary phase (45–63 h, OD_600_ from 3.961 to 4.320), the butanol synthesis accelerated, while both acetic acid and butyric acid decreased. In the decline period (63–84 h), acetic acid and butyric acid no longer changed, which was mainly due to the cell death and the formation of spore from the vegetative cell [8].

### Detection and identification of intracellular metabolites by metabolomics

Organic acids, such as acetic acid and butyric acid were produced along with the synthesis of ATP, and the subsequent production of butanol alleviated the toxicity from the organic acids. However, all these metabolites led to the fermentation degradation and finally terminated the cell growth, as shown in Fig. 1. Thus, it is pivotal to fully explore the optimal metabolic balance point between cell growth and butanol synthesis. From the insightful point of view, metabolic profiling analysis was carried out to explore their relationship. Metabolome sampling was implemented at 24, 48, 60 and 80 h, respectively, corresponding to the mid-log phase, the early and late stationary phase, and the decline phase of cell growth. At these time points, the specific growth rate μ were 0.0708, 0.0053, 0.0010, and 0.0031 (h^−1^), and the specific butanol production rate q were 0.0041, 0.0524, 0.0724 and 0.0004 (h^−1^). In this study, a total of 97 intracellular metabolites, mainly including sugars and their derivatives, amino acids, fatty acids and some other compounds had been detected, identified and quantified by GC-MS, MZ-mine 2.0 integrated with the NIST 2010 MS spectrum database.

**Fig. 2:**
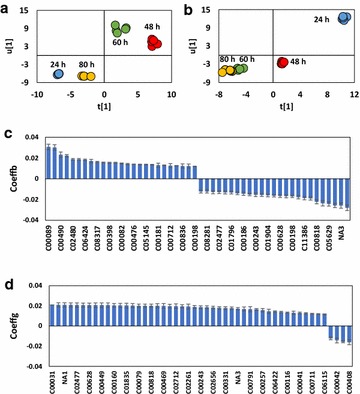
PLS modeling based on the metabolic profiling and specific butanol production rate/specific cell growth rate. **a** PLS score plot t[1]/u[1] for specific butanol production rate; **b** PLS score plot t[1]/u[1] for specific cell growth rate; **c** PLS coefficients plot for specific butanol production rate; **d** PLS coefficients plot for specific cell growth rate. All metabolites listed in the figure had a VIP greater than 1.0. “NA” stood for “not assigned” in the KEGG database. The complete metabolite list was given in the Supplement material. Coeffb, coefficient for specific butanol production rate; Coeffg, coefficient for specific growth rate

Subsequently, PLS was performed to evaluate the relationship between metabolites and cell growth/butanol production. The R^2^(X), R^2^(Y), and Q^2^ of PLS model were 0.832, 0.996, 0.994 for cell growth (μ), and 0.908, 0.964, 0.946 for butanol production (q), respectively, indicating the reliability of PLS analysis. Figure 2a, b highlighted the distinct diversity in butanol production and cell growth for different fermentation periods. Besides, in score plot, the tight clustering of five parallel samples and the clear separation of the sample types at each time point indicated that the metabolome data was acceptable for further analysis [17].

### Classification of metabolites and pathway identification with MetaboAnalyst 3.0

To further investigate the relationship between the intracellular metabolites and the extracellular phenotypes, variable importance in the projection (VIP) and coefficient of each metabolite were evaluated by the PLS model. Generally, metabolites with VIP value greater than 1.0 demonstrated a significant contribution within PLS model [18]. Therefore, these metabolites were displayed with KEGG number in Fig. 2c, d and Additional file 1: Table S1. Moreover, coefficient represented the impact value, i.e., positive coefficients correspond to the situation that the independent variable favors the target while the negative value for an opposite effect [19]. It was obvious that only 4 metabolites generated a negative effect on cell growth, but 26 metabolites went against the butanol production (Fig. 2c, d).

In this study, correlation of related metabolites were taken into consideration since no exclusive intracellular metabolite could affect the fermentation performance alone, especially, when a pathway was activated or inhibited, some related metabolites would behave collectively. Hence, it was necessary to classify these metabolites according to VIP and coefficient values. VIP value was given a priority to coefficient value for ensuring the significance of the target metabolites, thus 97 metabolites were mainly divided into 9 types (Table 1). Here, the eighth type (VIPb > 1 & VIPg > 1 & Coeffb > 0 & Coeffg > 0) and the ninth type (VIPb > 1 & VIPg > 1 & Coeffb < 0 & Coeffg < 0) were not displayed because both of them had no corresponding metabolites or pathways.

**Table 1:** Metabolic pathway classification and the corresponding metabolites

Type	Metabolic pathway	Metabolite
A VIPb < 1 VIPg < 1	Glutathione metabolism	Putrescine, glycine
		l-Glutamic acid
		Ascorbic acid
	Porphyrin and chlorophyll metabolism	l-Glutamic acid
		Glycine, l-Threonine
	Purine metabolism	Glycine, urea
		Deoxyguanosine
		Cyclic AMP
	Pyrimidine metabolism	Urea, malonic acid
		Deoxycytidine
		Methylmalonic acid
	Riboflavin metabolism	Riboflavin, ribitol
	Terpenoid backbone biosynthesis	Pyruvic acid
		Mevalonic acid
	Valine, leucine and isoleucine biosynthesis	Pyruvic acid, l-Valine l-Threonine
	Valine, leucine and isoleucine degradation	l-Valine
		Methylmalonic acid
B	Pentose phosphate pathway	Gluconolactone
VIPb > 1,Coeffb < 0	Glutathione metabolism	Pyroglutamic acid
VIPg < 1		
C	Fatty acid biosynthesis	Myristic acid
VIPb > 1, Coeffb > 0		Oleic acid, Stearic acid
VIPg < 1	Tryptophan metabolism	Tryptamine
		Oxoadipic acid
D	Amino sugar and nucleotide sugar metabolism	Glucose 1-phosphate d-Glucose, d-Mannose
VIPb > 1, Coeffb < 0	Galactose metabolism	d-Mannose, d-Glucose
VIPg > 1, Coeffg > 0		Glucose 1-phosphate
		Alpha-Lactose
	Glycolysis or gluconeogenesis	Glucose 1-phosphate
		d-Glucose, L-Lactic acid
	Pentose and glucuronate interconversions	Glucose 1-phosphate
		D-Arabitol
	Pentose phosphate pathway	d-Glucose, Gluconic acid
	Propanoate metabolism	L-Lactic acid
		2-Hydroxybutyric acid
	Starch and sucrose metabolism	d-Glucose
		Glucose 1-phosphate
	Pyruvate metabolism	L-Lactic acid
E	Alanine, aspartate and glutamate metabolism	l-Aspartic acid l-Alanine
VIPb < 1		Oxoglutaric acid
VIPg > 1, Coeffg > 0	Aminoacyl-tRNA biosynthesis	l-Phenylalanine
		l-Arginine, l-Serine
		l-Aspartic acid
		l-Alanine
	Cyanoamino acid metabolism	l-Serine, l-Aspartic acid
	Cysteine and methionine metabolism	l-Serine, l-Alanine
		l-Aspartic acid
	Glycine, serine and threonine metabolism	l-Serine, d-Serine
		l-Aspartic acid
	Glyoxylate and dicarboxylate metabolism	Glycolic acid, oxalic acid
		Oxoglutaric acid
	Histidine metabolism	Oxoglutaric acid
		l-Aspartic acid
F	Glyoxylate and dicarboxylate metabolism	Succinic acid
VIPb < 1	Butanoate metabolism	Succinic acid
VIPg > 1, Coeffg < 0	Phenylalanine metabolism	Succinic acid
	Alanine aspartate and glutamate metabolism	Succinic acid
	Citrate cycle (TCA cycle)	Succinic acid
	Lysine degradation	Pipecolic acid
	Propanoate metabolism	Succinic acid
	Tyrosine metabolism	Succinic acid
G	Aminoacyl-tRNA biosynthesis	l-Lysine
VIPb > 1, Coeffb > 0	Biotin metabolism	l-Lysine
VIPg > 1, Coeffg < 0	Lysine biosynthesis	l-Lysine
	Lysine degradation	l-Lysine

Subsequently, each type of metabolites were processed by the MetaboAnalyst 3.0 for metabolic pathway recognition (Table 1) [20]. This algorithm is based on over representation analysis and topology analysis [21, 22], which can be applied to evaluate the reliability (measured by p value) with Fisher’s exact test and the impact of each metabolic pathway by out-degree centrality separately. To ensure the pathway reliability, pathways with −log (p) > 1 were considered to be acceptable. Therefore, some metabolites and pathways were filtered. According to this principle, a total of 40 pathways and 43 metabolites were identified finally (Table 1). All the pathways and their corresponding −log (p) values and impact values are listed in Additional file 1: Table S2.

### Analysis of key metabolites and pathways associated with cell growth and butanol production

The key pathways and their corresponding metabolites would be summarized as follows.

**Fig. 3:**
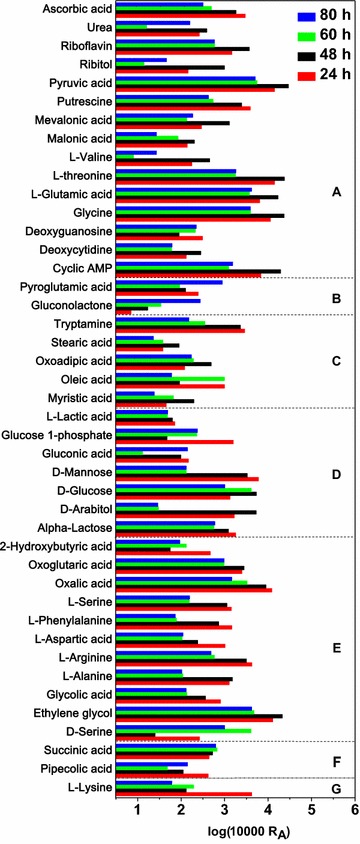
Relative abundance of the intracellular metabolites at each sample time. A total of 43 metabolites were divided into seven types and all the abundance values were expressed by log_10_(10,000*R_A_) function, where R_A_ was the relative abundance

As shown in Table 1, type A included glutathione metabolism, purine metabolism, pyrimidine metabolism, riboflavin metabolism, which were correlated with the primary cell growth metabolism. Their VIPs were less than 1.0, indicating that these pathways were insensitive factors for both cell growth and butanol production. Obviously, methylmalonic acid, l-threonine, pyruvic acid, glycine and l-glutamic acid accounted for the major abundance of the pathway (Fig. 3). Their abundance fell sharply from 48 to 60 h, which was corresponding to the decreased metabolic requirements from the early stationary phase to the late stationary phase, implying their importance on maintaining the normal cell metabolism.

**Fig. 4:**
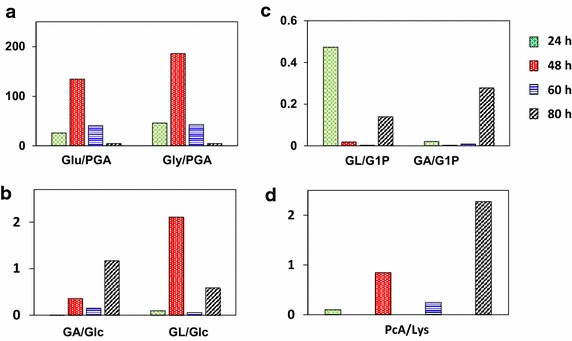
Ratio changes of closely related metabolites. Glu, l-glutamic acid; PGA, pyroglutamic acid; Gly, l-glycine; Glc, d-glucose; GA, gluconic acid; GL, gluconolactone; G1P, glucose 1-phosphote

Type B mainly exhibited a negative influence on butanol production (Table 1). Pyroglutamic acid (PGA), a dehydrated product of glutamic acid and correlated to the glutathione metabolism (Table 1), reached to the peak at the end of fermentation (Fig. 3). The ratio of glutamic acid/pyroglutamic acid (Glu/PGA) was 26, 134, 40 and 5, and the ratio of glycine/pyroglutamic acid (Gly/PGA) was 46, 186, 43 and 4, respectively (Fig. 4a). The downtrend of both ratios from early stationary phase to the end probably implied the accumulation of PGA and the decrease of glutathione biosynthesis, which further suppressed the butanol synthesis.

Gluconolactone (GL) is the first-step product of pentose phosphate (PP) pathway with NADP^+^ as the electron acceptor. The ratios of gluconic acid/glucose (GA/Glc) and gluconolactone/glucose (GL/Glc) maintained at a relatively high level at 24 h, then decreased, and finally increased at 80 h (Fig. 4b). At the same time, the changed pattern of gluconolactone/glucose 1-phosphote (GL/G1P) and gluconic acid/glucose 1-phosphote (GA/G1P) were similar to both GL/Glc and GA/Glc, although their first peaks appeared at 48 h (Fig. 4c). Both phenomena indicated that the oxidative PP pathway recovered at the later period of fermentation. Furthermore, gluconolactone seemingly contributed little to cell growth (Table 1, VIPg < 1) while the accumulation of gluconic acid was advantageous for cell growth, implying that PP pathway could be further strengthened for cell growth. However, both gluconolactone and gluconic acid were negatively associated with butanol production probably due to the mismatch of butanol dehydrogenase II (BDH II) with NADPH at the lower pH [23].

Type C contained fatty acid biosynthesis and tryptophan metabolism. The relative abundance of oleic acid (C_18_H_34_O_2_) and stearic acid (C_18_H_36_O_2_), initially declined at the early phase but then increased at the rapid butanol synthesis phase (Fig. 3), indicating a potential association with butanol synthesis. In addition, tryptamine and oxoadipic acid were correlated to the tryptophan metabolic pathway. Their relative abundance dropped rapidly by 85 and 61 % during the fast butanol synthesis period (after 48 h, Fig. 3). Their positive correlation to butanol production (VIPb > 1 and Coeffb > 0) suggested that on one hand, the increase of these two metabolites might promote butanol production, and on the other hand the consumption of tryptophan might be a response to the toxic stress of butanol [15, 24].

Type D consisted of several core sugar metabolic pathways (Table 1). l-lactic acid, glucose 1-phosphate, α-lactose and d-mannose fell off from the early stationary phase (48 h), while the intracellular d-glucose and d-arabitol initiated their decline from the late stationary phase (60 h) (Fig. 3). All the metabolites mentioned above were advantageous for cell growth (VIPg > 1 & Coeffg > 0) under our fermentation conditions, but disadvantageous for butanol production (VIPb > 1 & Coeffb < 0), suggesting that sugar metabolism was a key regulatory factor for the balance or trade-off between cell growth and butanol production. Interestingly, more than 20 kinds of sugar and their derivatives were detected in our samples (Additional file 1: Table S1), among them trehalose was outstanding as its abundance was 3.2–7.3 folds of d-glucose. Here, trehalose might protect the strain to resist osmotic stress throughout the fermentation [25, 26].

Type E was mainly involved in amino acid metabolism which was beneficial for cell growth but without significant effect on butanol production (VIPb < 1). Amino acids associated with methionine and cysteine metabolism (l-serine, l-aspartic acid) were depressed at the beginning of fermentation (48 h) (Fig. 3). Moreover, aminoacyl-tRNA biosynthesis pathway (l-phenylalanine, l-arginine, l-serine, l-aspartic acid) exhibited a downtrend from 24 to 80 h, indicating a shortage of protein synthesis. L-aspartate and glutamate metabolism (l-aspartic acid, l-alanine and oxoglutaric acid) had the highest impact value (0.31) on the metabolic network than any other pathway in Additional file 1: Table S2, implying their key role in the whole metabolic network. In addition, other metabolic pathways, such as histidine metabolism, cyanoamino acid metabolism, shared several metabolites with above pathways. All of them exhibited the similar effects on butanol production and cell growth. Moreover, it’s worth noting that intracellular abundance of oxalic acid as well as the ethylene glycol exceeded many other metabolites (Fig. 3), indicating that they played an important role in cell growth (VIPg > 1 & Coeffg > 0) as the intermediate metabolites of dicarboxylate (glyoxylate) metabolism (Table 1). Therefore, we postulated that the cell growth was partially limited by the poor availability of intracellular amino acids and oxalic acid or ethylene glycol, which can be strengthened by feeding experiments.

Type F consisted of only two metabolites, succinic acid and pipecolic acid, but they existed in eight pathways (Table 1). The relative abundance of succinic acid rose in the first stage, and then decreased, while pipecolic acid performed just the opposite, as shown in Fig. 3. Furthermore, succinic acid displayed a distinct difference with any other metabolites of the alanine, aspartate, glutamate and glyoxylate metabolic pathway in E-type, despite the fact that succinic acid belonged to these pathways mentioned (Table 1). Pipecolic acid was associated with lysine degradation pathway (Table 1). In Fig. 3 pipecolic acid (Type F) and l-lysine (type G) displayed the coincident behavior viewed from the degradation pathway. The ratio of pipecolic acid/l-lysine was 0.099, 0.848, 0.247 and 2.276, performing an uptrend of catabolism of l-lysine, especially at the end of fermentation (Fig. 4d). Both l-lysine and pipecolic acid were disadvantageous for cell growth while l-lysine was beneficial for butanol production by degradation metabolism (Table 1). The effects of succinic acid and lysine would be investigated in the following feeding experiment.

### Effects of rational addition of key metabolites on cell growth and butanol production

According to above analysis, threonine, glycine, phenylalanine, alanine, arginine, serine and aspartic acid were correlated to the normal cell metabolism, such as glutathione metabolism, purine metabolism, pyrimidine metabolism and aminoacyl-tRNA biosynthesis. At the same time, oxalic acid, ethylene glycol and succinic acid were also worth a further exploration for their complex effects on cell growth and butanol production. Thereby, these important metabolites including amino acids and organic acids associated with glyoxylate shunt and tricarboxylic acid (TCA) cycle, were exogenously fed into the fermentation medium to verify the previous pathway analysis and alleviate the production bottlenecks. In addition, histidine and valine were considered since they were involved in PP pathway and valine metabolic pathway, separately. Yeast extract was also added into the medium as a complex nitrogen source. Sugar metabolism was not considered to be further strengthened by feeding glucose because several studies had been carried out on fed-batch/continuous cultivation and genetic engineering previously [27–29]. They have confirmed that the glucose concentration should be kept at a proper level to reduce the substrate inhibition and overexpressing 6-phosphofructokinase and pyruvate kinase genes could effectively enhance the butanol production. In this study, all metabolites were added at the beginning of fermentation.

**Fig. 5:**
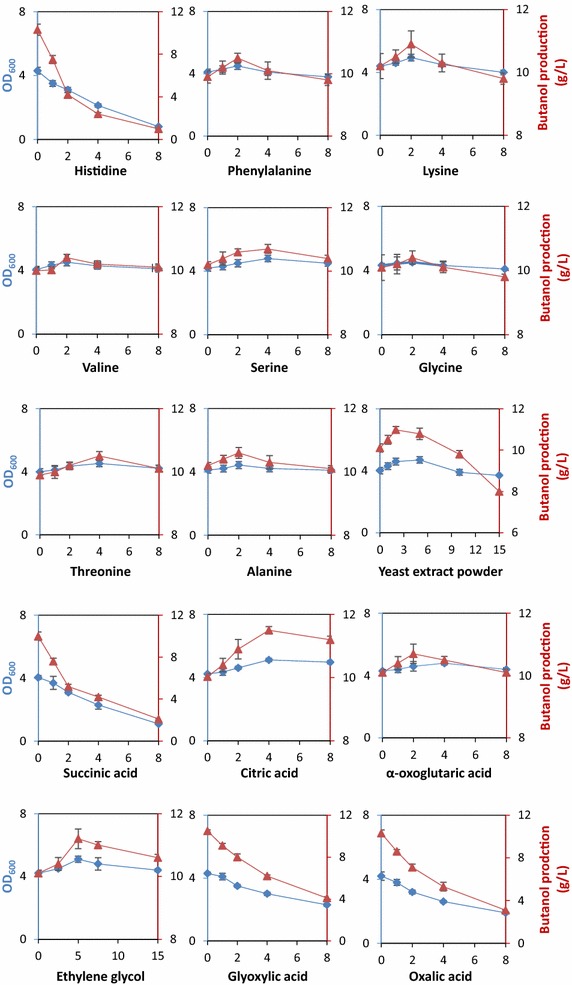
Effects of key metabolites addition on cell growth and butanol production. Values were the averages with standard deviations from three independent measurements. All the amino acids were L-configuration. The unit of horizontal ordinates was g/L, except for the ethylene glycol (mL/L). *Red triangles* stand for butanol production and *blue squares* stand for cell optical density at 600 nm (OD_600_)

As shown in Fig. 5, addition of most amino acids could increase the biomass. Among them feeding l-glycine and l-serine generated the highest cell biomass with the 13.9 and 14.3 % improvement, respectively, compared with the control. However, l-histidine addition failed to enhance the cell growth or butanol production, which was probably due to the inhibition of PP pathway as the end product. In addition, no amino acid could significantly increase butanol production in our study and all the increments were less than 5 %. Unexpectedly, the addition of l-lysine improved biomass by 12.3 %, which was inconsistent with the above analysis that all the lysine-related metabolic pathways had a negative effect on cell growth and butanol production. This phenomenon would be discussed in the Discuss section.

In addition, low concentration (<2 g/L) yeast extract exerted a positive effect on both cell growth and butanol production. However, when yeast extract was supplemented at the range of 2–5 g/L, cell growth continuously increased while butanol production changed slightly. Moreover, higher than 10 g/L of yeast extract inhibited both cell growth and butanol production. The highest butanol production of 10.98 ± 0.15 g/L was obtained at 2 g/L yeast extract. Therefore, it is necessary to control the yeast extract concentration in the feeding experiment.

Based on the analysis of dicarboxylate metabolism, as well as their relationships with butanol synthesis, various dicarboxylates were supplemented to improve intracellular dicarboxylates levels, which might promote more flux towards key precursors available for biomass synthesis and butanol biosynthesis. It’s notable that supplementation of ethylene glycol and citric acid resulted in an improvement on both cell growth and butanol production. In detail, 4 g/L citric acid increased the OD_600_ by 21.0 % and butanol production by 14.6 %, and 5 mL/L ethylene glycol generated 21.4 and 10.9 % increase of OD_600_ and butanol production, respectively, compared with the control. The highest butanol production was 11.20 ± 0.25 g/L for ethylene glycol and 11.5 ± 0.15 g/L for citric acid. Therefore, these results indicated that these intermediates were effectively utilized by feeding glyoxylate shunt (or TCA cycle) to enhance butanol titers. However, the feeding of oxalic acid, glycolic acid and glyoxylic acid failed to improve the butanol production. It was quite out of our expectation but it would not rule out the possibility of their chemical chelation with Fe^2+^ and Mn^2+^ in the medium, seriously depressing a series of enzymes.

Although succinic acid was a member of butyric acid synthesis pathway in KEGG database, supplementation of this compound displayed an inhibition effect on cell growth and butanol production (Fig. 5), indicating that the blindly reinforcement of butyric acid synthesis pathway just by empirical approach might lead to an opposite effect.

## Discussion

**Fig. 6:**
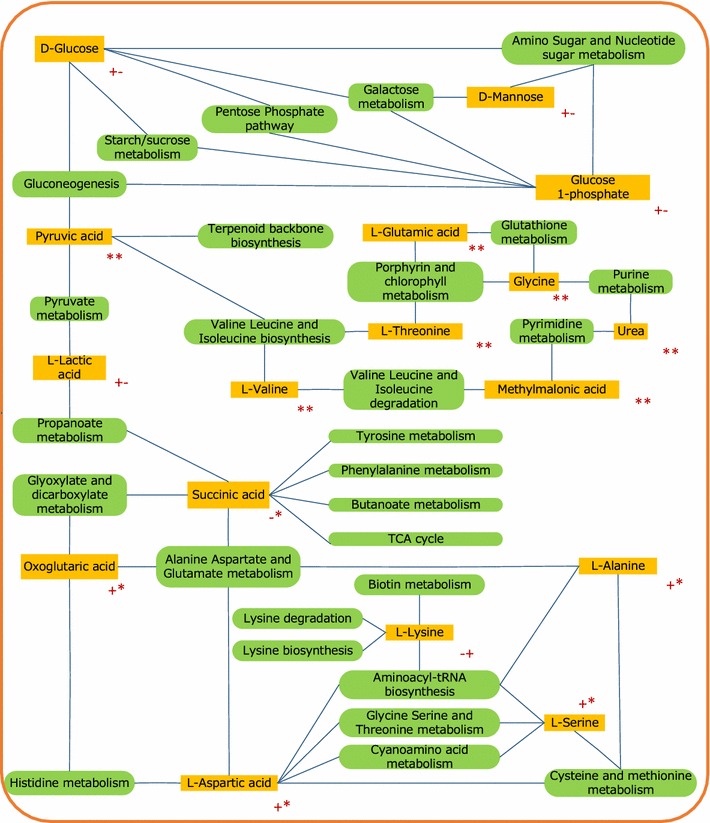
Metabolic network based on metabolic pathways linked with the key metabolites in our data analysis. *Yellow box* represents the key metabolites and the *green box* for the metabolic pathways. “+−”: VIPg > 1 & VIPb > 1 & Coeffg > 0 & Coeffb < 0; “**”: VIPg < 1 & VIPb < 1; “+*”: VIPg > 1 & VIPb < 1 & Coeffg > 0; “−*”: VIPg > 1 & VIPb < 1 & Coeffg < 0; “−+”: VIPg > 1 & VIPb > 1 & Coeffg < 0 & Coeffb > 0

In this study, metabolomics was applied to measure and evaluate the dynamic multiparametric metabolic responses of *C. acetobutylicum* to the inside and outside disturbance in a more comprehensive manner, thus enabled us to gain a more in-depth insight into complex mechanisms. In fact, the relationship between butanol synthesis and extracellular acetic acid, butyric acid, pH and residual sugar has been deeply studied previously, and many cues from acidogensis to solventogensis have been found [30–32]. However, these limited proofs could not offer more significant information about the intracellular metabolic activity and deeper insight into the mechanism. Recently, metabolomic analysis coupled with ^13^C isotopomer labelling of *C. acetobutylicum* has been employed to elucidate the metabolic characterization associated with butanol production [33–35], supplying a technical platform to comprehensively investigate the synthesis mechanism. Besides, principal component analysis and PLS modeling combined with metabolomics, have been successfully used for biomarker identification in *Streptomyces tsukubaensis* [36], *Rhizopus oryzae* [37], *Schizochytrium* sp. [38] and so on. In general, a metabolite with VIP > 1 could be usually considered as a biomarker which might be correlated with the target product. However, further explanation of a biomarker always falls into a confused condition since a metabolite frequently refers to several pathways. For example, both C00042 (succinic acid) and C00026 (α-oxoglutaric acid) were involved in TCA cycle, but their effects on the cell growth were opposite in our PLS model. Thus it is inappropriate to judge the impact of these biomarkers just by a simple combination of several compounds. In this work, we utilized the metabolic pathway analysis to get the p value and the pathway impact value to estimate the relationship between metabolites and pathways, and finally found that succinic acid was the pathway node of TCA cycle, tyrosine metabolism, phenylalanine metabolism and other four metabolic pathways (Fig. 6). Therefore, the abundance of succinic acid indicated the net accumulation of these pathways, not merely the TCA cycle, which avoided the incorrect empirical judgment.

From the viewpoint of precursors to products, intracellular metabolite abundance represents a potential, or an indispensable power driving the metabolic reaction to the target product. Redundancy or shortage of crucial metabolites could change the profiles of cell metabolism since a certain metabolite often participates in several metabolic pathways. In metabolic profiling, metabolites are also the nodes of several related metabolic pathways, just as shown in Fig. 6. For this reason, pathway analysis, or “metabolite set analysis” can generally offer more metabolic mechanism than biomarker analysis.

With regard to the sugar metabolism, glucose was used as the sole carbon source in our study, but maltose, trehalose, sorbose, sucrose and other 20 kinds of sugar and their derivatives were also detected by GC-MS, indicating that the catabolism rate of glucose was restricted under our experiment condition (initial glucose concentration was set at 50.0 g/L), leading to the accumulation of other sugars. As the core metabolism, sugar conversion and catabolism provide ATP and NAD(P)H for both cell growth and shift signal from acidogensis to solventogensis [6]. In our PLS model, sugar metabolism was crucial for cell growth but went against butanol synthesis. As we known, the products of glucose catabolism would cause the acidification of medium, even the “acid crash effect” through the synthesis of abundant acetic acid and butyric acid [7]. Therefore, low intracellular pH results in a significant transmembrane proton gradient, which consumes ATP to maintain the normal physiological status. The butanol synthesis is considered as a detoxication process by uptake of acetic acid and butyric acid [39] at the expense of cell membrane damage and cell construction instability [15, 40]. Here, strain needs a balance between cell survival and butanol production by regulating sugar metabolism. In addition, a fed-batch or pH-control system is usually adopted to maintain sugar metabolism at a proper level for protecting the cell from acid crash and improve butanol production [41, 42]. Thereby, sugar metabolism is a regulatory target for both cell growth and butanol production.

According to the metabolic profiling analysis, we should take more attention to the accumulation of oxalic acid (C_2_H_2_O_4_) and its reductive product ethylene glycol (C_2_H_6_O_2_). Up to now, both glyoxylate and dicarboxylate metabolism have been poorly investigated in *C. acetobutylicum*. As shown in Fig. 6, glyoxylate and dicarboxylate metabolism is connected with seven pathways via succinic acid and α-oxoglutaric acid. In this study, both citric acid and ethylene glycol had successfully promoted the cell growth and butanol production probably by strengthening the glyoxylate and dicarboxylate metabolism, suggesting that excess C2-compounds could be converted to both cell building blocks and butanol precursors.

The changes of succinic acid and lysine seemed non-associated with other metabolites of the same pathway in this study. In fact, previous study has revealed that both ^13^C-aspartate and ^13^C-glutamate could produce ^13^C-labeled succinic acid, and succinyl-CoA flux into succinic acid was about 25 %, while the methionine combined with lysine biosynthesis pathways contributed to the rest of the total flux [34]. Furthermore, it has been reported that in TCA cycle, metabolic flux has not been measured for the steps between α-ketoglutarate and succinyl-CoA, succinic acid and fumarate, malate and oxaloacetate [43]. In our study, succinic acid achieved the peak at 60 h in keeping with the recovery of l-lysine, confirming the interconversion between succinic acid and lysine. Therefore, in the feeding experiments addition of lysine could promote cell growth probably due to the reason that lysine was used for biomass synthesis in the beginning and excess succinic acid was converted into lysine at the end of the fermentation. Considering the fact that glyoxylate shunt could partly replace TCA cycle, glyoxylate shunt played a more important role for *C. acetobutylicum* rather than TCA cycle. Thereby, the interconversion of lysine, and succinic acid and the C2 metabolism provided valuable insights into the metabolism of *C. acetobutylicum*.

Another significant aspect to be considered was the accumulation of amino acids. As shown in Fig. 6, among the 17 key metabolic nodes, amino acids accounted for approximately one half. Most of the amino acids and the yeast extract have been proved to be helpful for cell growth, in agreement with previous work [44]. Generally speaking, *C. acetobutylicum* could utilize nitrogen-limited media to synthesize the building blocks for cell growth as well as butanol production. In this study, the nitrogen source in initial P2 medium was ammonium acetate, ammonium hydroxide and a low-concentration complex nitrogen source from the reinforced clostridium medium (RCM). Superabundant nitrogen source could promote cell growth, but would not result in more butanol in these media since nourishing media might be not beneficial for the initiation of butanol synthesis, such as the conditions of *Clostridium* growth medium, bromcresol purple dextrose peptone medium and the RCM medium, in which organic acids were the main products. Therefore, most of amino acids could significantly improve the cell density rather than butanol production.

For single amino acid, both glycine and glutamate were correlated to the glutathione metabolism as described in Results section. It should be noted that glutathione plays an important role in the anti-oxidative process [45] and the adaptation to butanol stress [46]. In this study, both l-glycine and l-glutamic acid were abundant, in accordance with the previous study [33], ensuring the synthesis of glutathione to protect cell from the oxidative stress and strengthen the robustness of *C. acetobutylicum* under the butanol and organic acids stress.

The last notable feature was the fatty acid biosynthesis, which plays an important role in cell membrane system. The cell membrane fluidity can be adjusted by regulating the length and unsaturation degree to resist the butanol stress from acidogenesis to solventogensis, which has been deeply reported in the previous works [14, 47]. In this study, the key metabolites referring to fatty acid biosynthesis pathway (oleic acid and stearic acid) recovered to the high level when butanol production was at the highest rate, which was consistent with above response mechanism.

From the PLS model and pathway analysis, two types of metabolic pathway based on over representation analysis and pathway topology analysis were not considered. One case was that no metabolites could simultaneously satisfy the condition: VIPb > 1 & VIPg > 1 & Coeffb > 0 & Coeffg > 0. It meant that there was no metabolite that could strengthen both cell growth and butanol production under our fermentation condition. Another phenomenon was that only erythrose met the condition: VIPb > 1 & VIPg > 1 & Coeffb < 0 & Coeffg < 0. Unfortunately, erythrose cannot be divided into any pathways, which was out of our expectation. Thus, there was no metabolite that was excess for both cell growth and butanol in our study. However, our experiments have verified that feeding of succinic acid and l-histidine inhibited both cell growth and butanol production, and ethylene glycol and citric acid promoted both cell growth and butanol production, suggesting a more sophisticated metabolic regulation for *C. acetobutylicum*.

## Conclusions

Based on in-depth analysis of key metabolites and pathways, rational feeding strategies of these potential metabolites were proposed to direct the improvement of cell growth and butanol production. From a practical point of view, these strategies can also be extended to strengthen biosynthesis of other important microbial natural products and process optimization. Besides, it is necessary to combine in silico genome-scale model with metabolomics and genetic engineering, which will reveal more complicated metabolic mechanisms and form a more powerful tool for the titer improvement in the future.

## Methods

### Strain and medium

*Clostridium acetobutylicum* ATCC 824 obtained from the American type center culture collection (ATCC, Maryland, USA) was stored with 20–30 % glycerin-water solution at −80 °C.

RCM was used for seed culture: 3 g/L yeast extract, 10 g/L beef extract, 10 g/L peptone, 1.0 g/L soluble starch, 5 g/L glucose, 0.5 g/L l-cysteine hydrochloride, 3 g/L NaCl, 3 g/L NaAc, 3 mg/L resazurin sodium, pH 7.0.

P2 culture was used for fermentation: 50 g/L glucose, 0.5 g/L K_2_HPO_4_, 2.2 g/L ammonium acetate, 0.5 g/L KH_2_PO_4_, 0.2 g/L MgSO_4_·7H_2_O, 0.01 g/L FeSO_4_·7H_2_O, 0.01 g/L NaCl, 0.01 g/L MnSO_4_·H_2_O, 1 mg/L amino benzoic acid, 1 mg/L vitamin B_1_ and 0.001 mg/L D-biotin, pH 6.4 ~ 6.6. The media were sterilized at 115 °C for 20 min.

### Cultivation conditions

Spore suspension was heat-shocked at 85 °C for 15 min, then quickly put into cold water (<4 °C) for 15 min before inoculation.

YQX-II anaerobic chamber (Shanghai LongYue instrument Co., LTD, China) was used for anaerobic cultivation by purging with mixed gas (N_2_:H_2_:CO_2_ = 85:10:5, purity >99.9 %). Seed medium was maintained in the anaerobic chamber for more than 12 h to eliminate the remaining oxygen. Then 1 mL spore suspension was inoculated into 50 mL seed medium and cultivated at 37 °C for another 24 h.

Anaerobic fermentation operation was carried out in a 3.5 L fermentation tank (NBS BIOFLO2000 New Brunswick Scientific, USA), with a 1.8 L working volume. Before inoculation, 0.1 L per minute (LPM) sterile mixed gas (N_2_:H_2_:CO_2_ = 85:10:5, purity 99.9 %) was bubbled into fermentation culture for 60 min and then inoculated with 5 % seed media. Subsequently, the sterile mixed gas rate was set at 0.01 LPM. The initial pH was adjusted to 6.8 by 6 M ammonia and monitored by pH meter. The temperature and stirring rate were maintained at 37 °C and 100 rpm, respectively.

### Analytical methods

Biomass was monitored by measuring the optical density. Generally, 1 mL of fermentation broth was sampled to determine OD_600_ using UV spectrophotometer (ZF1-II, Shanghai JiaPeng technology, China) after an appropriate dilution. The concentration of acetic acid, butyric acid and butanol were detected by gas chromatography (GC) (Bruker Daltonics Inc, Germany) equipped with BR-S Wax capillary column (30 m, 0.32 mm ID, 0.25 microns) and FID detector. Fermentation pH was measured by pH electrode (PB-10, Sartorius scientific instruments co, ltd, Germany). Residual glucose concentration in the fermentation broth was detected by biosensor analyzer SBA-40C (Academy of Sciences Institute of Shandong province, China). Each experiment was detected by at least triple biological replicates.

### Detection of intracellular metabolites based on gas chromatography-mass spectrometry (GC-MS)

Sample preparation was conducted by a modified two-step protocol: quenching-extraction and derivation under the atmospheric condition [35, 48].

In the first step, all the operation should be kept under a low temperature condition. 10 mL fermentation broth was collected and immediately quenched by 20 mL, −20 °C 60 % methanol solution to terminate cell physiological metabolism, followed by centrifugation at 3000 rpm, 4 °C for 3 min. Precipitate was washed by phosphate buffered solution (PBS) for three times at 4 °C and re-suspended with 1.5 mL of −40 °C 50 % (v/v) methanol solution, then thawed in an ice bath for 5 min. The mixture was blended with a vortex mixer for 20 s, and put into the liquid nitrogen to be re-freezed for 5 min and thawed for 1 min at −20 °C. Five times freezing-thawing operation was carried out and the supernatant was collected and blended with 50 µL succinic acid-2,2,3,3-d4 (CAS: 14493-42-6, 0.3 mg/mL or 230 µM) as internal standard, frozen drying for 8 h at −45 °C, then stored at −80 °C.

Derivatization was used for lowering the polarity of the compounds and gasification temperature to expand the spectrum of GC-MS [49]. Here, we adopted the silane derivatization approach. In detail, 50 µL of 20 mg/mL methoxylamine hydrochloride—pyridine solution was added into the sample, then kept at 40 °C for 90 min. Subsequently, 80 µL *N*-methyl-*N*-(trimethylsilyl) trifluoroacetamide was added and mixed, later kept at 40 °C for 30 min.

Data acquisition by GC-MS was performed by the system including A7683B automatic sampler, GC analyzer 6890 N and 5975C MSD mass spectrometer (Agilent Technologies, Inc., USA) equipped with Agilent HP-5MS capillary column. GC parameters: sample quantity 1 µL; split ratio 1:1; injection port temperature 280 °C; interface temperature 280 °C; helium gas velocity, constant pressure of 91 kpa, 1.0 mL/min. Temperature program: 70 °C for 2 min; 5 °C/min up to 290 °C, holding for 3 min. Mass spectrometer parameters: electron impact (EI+) ion source; 250 °C; electron bombardment energy 70 eV; ion current 40 µA; scan range of mass: 50–650 m/z.

### Quantitative calculation method

MS data was processed with the Agilent MSD Productivity ChemStation (Agilent Technologies Inc. USA), and MZ-2.10 (http://MZmine.sourceforge.net/) for MS spectrum deconvolution, denoising, retention time aligning, peak area integration and compound identification combined with NIST 2010 mass spectrum database (http://webbook.nist.gov/chemistry/). All the identified compounds should match the KEGG compounds after substituting the trimethylsilane (-TMS) type by hydrogen atom.

Relative abundance of each metabolite was defined as A_r_. The value of A_r_, the specific butanol production rate q, and the specific cell growth rate µ could be calculated from the following formulas.

1 $${\text{A}}_{r} = \frac{\text{Ai}}{{{\text{DCW}} \cdot {\text{As}}}}$$

2 $${\text{q}} = \frac{\text{dc}}{{{\text{DCW}} \cdot {\text{dt}}}}$$

3 $$\upmu = \frac{\text{dlnOD}}{{ {\text{dt}}}}$$

where A_i_: measured value of each peak area; As: area of succinic acid-2,2,3,3-d4; DCW: drying cell weight; c: butanol concentration.

PLS, a supervised multivariate analysis, was conducted by SIMCA-p 11.5 (Umetrics AB, Sweden, Windows environment) to investigate the relationship between intracellular metabolites and extracellular phenotypes (µ and q).

## Additional file

10.1186/s12934-016-0436-4 All the metabolites detected in our study and the results of pathway analysis.

## Abbreviations

ABE: acetone—butanol—ethanol
PCA: principal component analysis
PLS: partial least square
TCA: tricarboxylic acid
RCM: reinforced clostridium medium
GC-MS: gas chromatography-mass spectrometry
VIP: variable importance in the projection
VIPb: VIP for specific butanol production rate
VIPg: VIP for specific growth rate
Coeffb: coefficient for specific butanol production rate
Coeffg: coefficient for specific growth rate
Glu: l-glutamic acid
PGA: pyroglutamic acid
Gly: l-glycine
Glc: d-glucose
GA: gluconic acid
GL: gluconolactone
G1P: glucose 1-phosphote
