# Supplementary material for: Integrated intracellular metabolic profiling and pathway analysis approaches reveal complex metabolic regulation by Clostridium acetobutylicum
Source: Microb Cell Fact. 2016 Feb 15;15:36. doi: 10.1186/s12934-016-0436-4 (PMC4753663; doi:10.1186/s12934-016-0436-4)
Supplement: Supplementary file 1 — 10.1186/s12934-016-0436-4 All the metabolites detected in our study and the results of pathway analysis. [file 12934_2016_436_MOESM1_ESM.docx]

Integrated intracellular metabolic profiling and pathway analysis approaches reveal complex metabolic regulation by ***Clostridium acetobutylicum***

Huanhuan Liu^a,b,1^, Di Huang^c,d,e,1^, Jianping Wen^a,b^ ^*^

^a^Key Laboratory of System Bioengineering (Tianjin University), Ministry of Education, Tianjin, 300072, People’s Republic of China

^b^SynBio Research Platform, Collaborative Innovation Center of Chemical Science and Engineering (Tianjin), School of Chemical Engineering and Technology, Tianjin University, Tianjin, 300072, People’s Republic of China

^c^TEDA Institute of Biological Sciences and Biotechnology, Nankai University, TEDA, Tianjin 300457, People’s Republic of China

^d^Key Laboratory of Molecular Microbiology and Technology, Ministry of Education, Tianjin 300071, People’s Republic of China

^e^Tianjin Key Laboratory of Microbial Functional Genomics, Tianjin 300457, People’s Republic of China

^1^These authors contributed equally to this work.

^*^Corresponding author

E-mail addresses:

HL: liuhuan@tju.edu.cn

DH: huangdi@nankai.edu. cn

JW: [jpwen@tju.edu.cn](mailto:jpwen@tju.edu.cn)

**Table S1** KEGG No. and relative abundance for each identified metabolite from cultures at different time points

| Hit | KEGG No. | Relative abundance^a^ | | | |
| --- | --- | --- | --- | --- | --- |
|  |  | 24 h | 48 h | 60 h | 80 h |
| 2-Hydroxybutyric acid | C05984 | 0.0470 | 0.0056 | 0.0132 | 0.0093 |
| 3-Aminoisobutanoic acid | C05145 | 0.8945 | 2.0694 | 0.6758 | 0.2501 |
| 3-Hydroxybutyric acid | C01089 | 5.9495 | 10.4801 | 1.9284 | 1.9036 |
| 4-Hydroxyproline | C01157 | 0.0272 | 0.0000 | 0.0996 | 0.0730 |
| ADP | C00008 | 0.0197 | 0.5627 | 0.0022 | 0.0022 |
| Allose | C01487 | 0.0996 | 0.1726 | 0.0371 | 0.0377 |
| Alpha-Lactose | C00243 | 0.1799 | 0.1229 | 0.0570 | 0.0595 |
| Alpha-Tocopherol | C02477 | 0.0528 | 0.0199 | 0.0098 | 0.0034 |
| Ascorbic acid | C00072 | 0.2963 | 0.1827 | 0.0495 | 0.0319 |
| Behenic acid | C08281 | 0.0549 | 0.0407 | 0.0100 | 0.0130 |
| Butyric acid | C00246 | 0.0025 | 0.4099 | 0.1251 | 0.1359 |
| Cellobiose | C06422 | 0.0805 | 0.1750 | 0.0352 | 0.0357 |
| cis,cis-Muconic acid | C02480 | 0.0170 | 0.0808 | 0.0329 | 0.0250 |
| cis-Aconitic acid | C00417 | 0.4064 | 0.2913 | 0.0826 | 0.0859 |
| Citric acid | C00158 | 0.0189 | 0.3959 | 0.0030 | 0.0030 |
| Creatinine | C00791 | 0.0179 | 0.0182 | 0.0051 | 0.0053 |
| Cyclic AMP | C00575 | 0.6795 | 1.9423 | 0.1234 | 0.1536 |
| D-(+)-Ribono-1,4-lactone | - | 0.0350 | 0.5404 | 0.0009 | 0.0015 |
| D-Alpha-aminobutyric acid | C02261 | 0.1936 | 0.1046 | 0.0476 | 0.0486 |
| D-Arabitol | C01904 | 0.1660 | 0.5318 | 0.0030 | 0.0029 |
| Deoxycytidine | C00881 | 0.0130 | 0.0283 | 0.0060 | 0.0062 |
| Deoxyguanosine | C00330 | 0.0310 | 0.0090 | 0.0213 | 0.0222 |
| D-Galactose | C00124 | 0.1724 | 0.0654 | 0.0164 | 0.0055 |
| D-Glucaric acid | C00818 | 0.0493 | 0.0037 | 0.0188 | 0.0195 |
| D-Glucose | C00031 | 0.1332 | 0.5362 | 0.4092 | 0.1015 |
| D-Lyxose | C08348 | 0.0210 | 0.0944 | 0.0134 | 0.0994 |
| D-Maltose | C00208 | 0.1151 | 0.3255 | 0.0406 | 0.0208 |
| D-Mannose | C00159 | 0.5989 | 0.3308 | 0.0131 | 0.0132 |
| D-Mannose 1-phosphate | C00636 | 0.0057 | 0.5435 | 0.0014 | 0.0014 |
| D-Ornithine | C00515 | 0.0014 | 0.0992 | 0.0004 | 0.0004 |
| D-Serine | C00740 | 0.0265 | 0.0025 | 0.4037 | 0.0995 |
| D-Tagatose | C00795 | 0.0000 | 0.0992 | 0.0007 | 0.0008 |
| D-Xylose | C00181 | 0.0031 | 0.4023 | 0.0006 | 0.0007 |
| Erythrose | C01796 | 0.4210 | 0.0005 | 0.0070 | 0.0072 |
| Ethanol | C00469 | 0.0141 | 0.0082 | 0.0010 | 0.0010 |
| Ethylene glycol | C01380 | 1.2805 | 2.1211 | 0.4726 | 0.4199 |
| Gentisic acid | C00628 | 0.1931 | 0.0017 | 0.0034 | 0.0009 |
| Gluconic acid | C00257 | 0.0147 | 0.0099 | 0.0013 | 0.0139 |
| Gluconolactone | C00198 | 0.0007 | 0.0017 | 0.0034 | 0.0276 |
| Glucose 1-phosphate | C00103 | 0.1579 | 0.0047 | 0.0229 | 0.0236 |
| Glycerol | C00116 | 0.0108 | 0.0113 | 0.0039 | 0.0038 |
| Glycine | C00037 | 1.1346 | 2.3404 | 0.3942 | 0.3915 |
| Glycolic acid | C00160 | 0.0805 | 0.0362 | 0.0134 | 0.0130 |
| Hydrocinnamic acid | C05629 | 0.0660 | 0.0192 | 0.0095 | 0.0327 |
| Indolepyruvate | C00331 | 0.0109 | 0.0098 | 0.0029 | 0.0030 |
| Isocitric acid | C00311 | 0.0122 | 0.0540 | 0.4002 | 0.0992 |
| Itaconic acid | C00490 | 0.0156 | 0.0119 | 0.0526 | 0.0505 |
| L-Alanine | C00041 | 0.1270 | 0.1501 | 0.0109 | 0.0103 |
| L-Arginine | C00062 | 0.4230 | 0.3160 | 0.0582 | 0.0487 |
| L-Asparagine | C00152 | 0.0385 | 0.0631 | 0.0117 | 0.0119 |
| L-Aspartic acid | C00049 | 0.1018 | 0.0240 | 0.0106 | 0.0110 |
| L-Citronellol | C11386 | 0.3467 | 0.0020 | 0.0005 | 0.0080 |
| L-Glutamic acid | C00025 | 0.6418 | 1.6916 | 0.3722 | 0.4176 |
| L-Histidine | C00135 | 0.0154 | 0.0226 | 0.0070 | 0.0069 |
| L-Homoserine | C00263 | 0.4886 | 0.5979 | 0.1163 | 0.1090 |
| L-Lactic acid | C00186 | 0.0071 | 0.0063 | 0.0048 | 0.0049 |
| L-Lysine | C00047 | 0.4210 | 0.0131 | 0.0194 | 0.0061 |
| L-Phenylalanine | C00079 | 0.1460 | 0.0731 | 0.0078 | 0.0073 |
| L-Proline | C00148 | 0.0038 | 0.0039 | 0.0072 | 0.0075 |
| L-Serine | C00065 | 0.1418 | 0.1124 | 0.0154 | 0.0156 |
| L-Threonine | C00188 | 1.4031 | 2.3703 | 0.1770 | 0.1810 |
| L-Tyrosine | C00082 | 0.4211 | 0.0329 | 0.0073 | 0.0082 |
| L-Valine | C00183 | 0.0177 | 0.0451 | 0.0008 | 0.0027 |
| Malic acid | C00711 | 0.0015 | 0.4997 | 0.4001 | 0.0992 |
| Malonic acid | C00383 | 0.0137 | 0.0201 | 0.0085 | 0.0027 |
| Maltotriose | C01835 | 0.0429 | 0.0147 | 0.0164 | 0.0037 |
| Mannobiose | C01728 | 0.0893 | 0.1505 | 0.0486 | 0.0669 |
| Mannose 6-phosphate | C00275 | 0.0039 | 0.0992 | 0.0018 | 0.0018 |
| Methylmalonic acid | C02170 | 1.7615 | 7.4298 | 0.2881 | 0.4302 |
| Mevalonic acid | C00418 | 0.0295 | 0.1287 | 0.0134 | 0.0184 |
| Myristic acid | C06424 | 0.0045 | 0.0196 | 0.0066 | 0.0024 |
| N-Acetyl-L-methionine | C02712 | 0.1239 | 0.0645 | 0.0007 | 0.0039 |
| Oleic acid | C00712 | 0.0992 | 0.0091 | 0.0992 | 0.0060 |
| Oxalic acid | C00209 | 1.2151 | 0.8907 | 0.3297 | 0.1460 |
| Oxoadipic acid | C00322 | 0.0121 | 0.0490 | 0.0191 | 0.0172 |
| Oxoglutaric acid | C00026 | 0.2480 | 0.2814 | 0.0968 | 0.0979 |
| Palmitic acid | C00249 | 0.0139 | 0.0511 | 0.0011 | 0.0060 |
| Pentadecanoic acid | C16537 | 0.0299 | 0.0320 | 0.0369 | 0.0231 |
| Pimelic acid | C02656 | 0.0500 | 0.0377 | 0.0028 | 0.0043 |
| Pipecolic acid | C00408 | 0.0417 | 0.0111 | 0.0048 | 0.0139 |
| Putrescine | C00134 | 0.3882 | 0.2462 | 0.0543 | 0.0423 |
| Pyrimidine | C00396 | 0.0223 | 0.0537 | 0.0020 | 0.0067 |
| Pyroglutamic acid | C01879 | 0.0246 | 0.0126 | 0.0092 | 0.0885 |
| Pyruvic acid | C00022 | 1.4029 | 2.9444 | 0.5543 | 0.5072 |
| Rhamnose | C00507 | 0.1324 | 0.2034 | 0.0302 | 0.0286 |
| Ribitol | C00474 | 0.0144 | 0.0992 | 0.0014 | 0.0046 |
| Riboflavin | C00255 | 0.1474 | 0.3690 | 0.0592 | 0.0583 |
| Saccharopine | C00449 | 0.0752 | 0.0197 | 0.0246 | 0.0160 |
| Sedoheptulose | C02076 | 0.1076 | 0.0377 | 0.0121 | 0.0219 |
| Sorbitol | C00794 | 0.0378 | 0.0992 | 0.0064 | 0.0066 |
| Sphinganine | C00836 | 0.0054 | 0.4017 | 0.0029 | 0.0030 |
| Stearic acid | C01530 | 0.0038 | 0.0090 | 0.0038 | 0.0023 |
| Succinic acid | C00042 | 0.0439 | 0.0525 | 0.0671 | 0.0621 |
| Sucrose | C00089 | 0.0330 | 0.6266 | 0.0182 | 0.0355 |
| Trehalose | C01083 | 0.9758 | 1.8790 | 0.2759 | 0.3246 |
| Tryptamine | C00398 | 0.2895 | 0.2317 | 0.0353 | 0.0151 |
| Urea | C00086 | 0.0260 | 0.0388 | 0.0016 | 0.0159 |

- not assigned

^a^ The values represent the means of five independent experiments

Table S2 Metabolic pathway classification based on over representation analysis and pathway topology analysis

| Type | Metabolic pathway | -log(p) | Impact |  |
| --- | --- | --- | --- | --- |
| A | Valine, leucine and isoleucine biosynthesis | 1.56 | 0.15 |  |
|  | Porphyrin and chlorophyll metabolism | 1.56 | 0.03 |  |
|  | Glutathione metabolism | 1.11 | 0.06 |  |
|  | Purine metabolism | 1.11 | 0.04 |  |
|  | Pyrimidine metabolism | 1.11 | 0.02 |  |
|  | Riboflavin metabolism | 1.03 | 0.12 |  |
|  | Terpenoid backbone biosynthesis | 1.03 | 0.08 |  |
|  | Valine, leucine and isoleucine degradation | 1.03 | 0.02 |  |
| B | Pentose phosphate pathway | 2.17 | 0.02 |  |
|  | Glutathione metabolism | 1.96 | 0.02 |  |
| C | Fatty acid biosynthesis | 1.67 | 0 |  |
|  | Tryptophan metabolism | 1.16 | 0.05 |  |
| D | Glycolysis or Gluconeogenesis | 3.29 | 0.09 |  |
|  | Galactose metabolism | 3.01 | 0.09 |  |
|  | Amino sugar and nucleotide sugar metabolism | 2.71 | 0.05 |  |
|  | Pentose phosphate pathway | 1.95 | 0.09 |  |
|  | Propanoate metabolism | 1.55 | 0.04 |  |
|  | Starch and sucrose metabolism | 1.25 | 0.1 |  |
|  | Pyruvate metabolism | 1.13 | 0.05 |  |
|  | Pentose and glucuronate interconversions | 1.03 | 0.03 |  |
| E | Cysteine and methionine metabolism | 1.96 | 0.05 |  |
|  | Aminoacyl-tRNA biosynthesis | 1.6 | 0.11 |  |
|  | Alanine, aspartate and glutamate metabolism | 1.57 | 0.31 |  |
|  | Histidine metabolism | 1.45 | 0 |  |
|  | Cyanoamino acid metabolism | 1.45 | 0.08 |  |
|  | Glycine, serine and threonine metabolism | 1.27 | 0.19 |  |
|  | Glyoxylate and dicarboxylate metabolism | 1.03 | 0.08 |  |
| F | Tyrosine metabolism | 2.34 | 0 |  |
|  | Propanoate metabolism | 2.34 | 0.02 |  |
|  | Lysine degradation | 2.17 | 0.02 |  |
|  | Citrate cycle (TCA cycle) | 2.17 | 0.06 |  |
|  | Alanine, aspartate and glutamate metabolism | 2.02 | 0 |  |
|  | Phenylalanine metabolism | 2.02 | 0 |  |
|  | Butanoate metabolism | 2.02 | 0.02 |  |
|  | Glyoxylate and dicarboxylate metabolism | 1.78 | 0 |  |
| G | Biotin metabolism | 3.53 | 0 |  |
|  | Lysine degradation | 2.83 | 0.16 | |
|  | Lysine biosynthesis | 2.55 | 0.03 | |
|  | Aminoacyl-tRNA biosynthesis | 1.99 | 0.03 | |

^a^ p value represented the hit reliability of a pathway based on the over representation analysis with Fisher's exact test and the impact value indicated the centrality of the out-degree from pathway topology analysis.
